# Supplementary material for: Porous translucent electrodes enhance current generation from photosynthetic biofilms
Source: Nat Commun. 2018 Apr 3;9:1299. doi: 10.1038/s41467-018-03320-x (PMC5880806; doi:10.1038/s41467-018-03320-x)
Supplement: Supplementary file 1 — Supplementary Information [file 41467_2018_3320_MOESM1_ESM.pdf]

Porous translucent electrodes enhance current generation from photosynthetic biofilms

Tobias Wenzel *et al.*

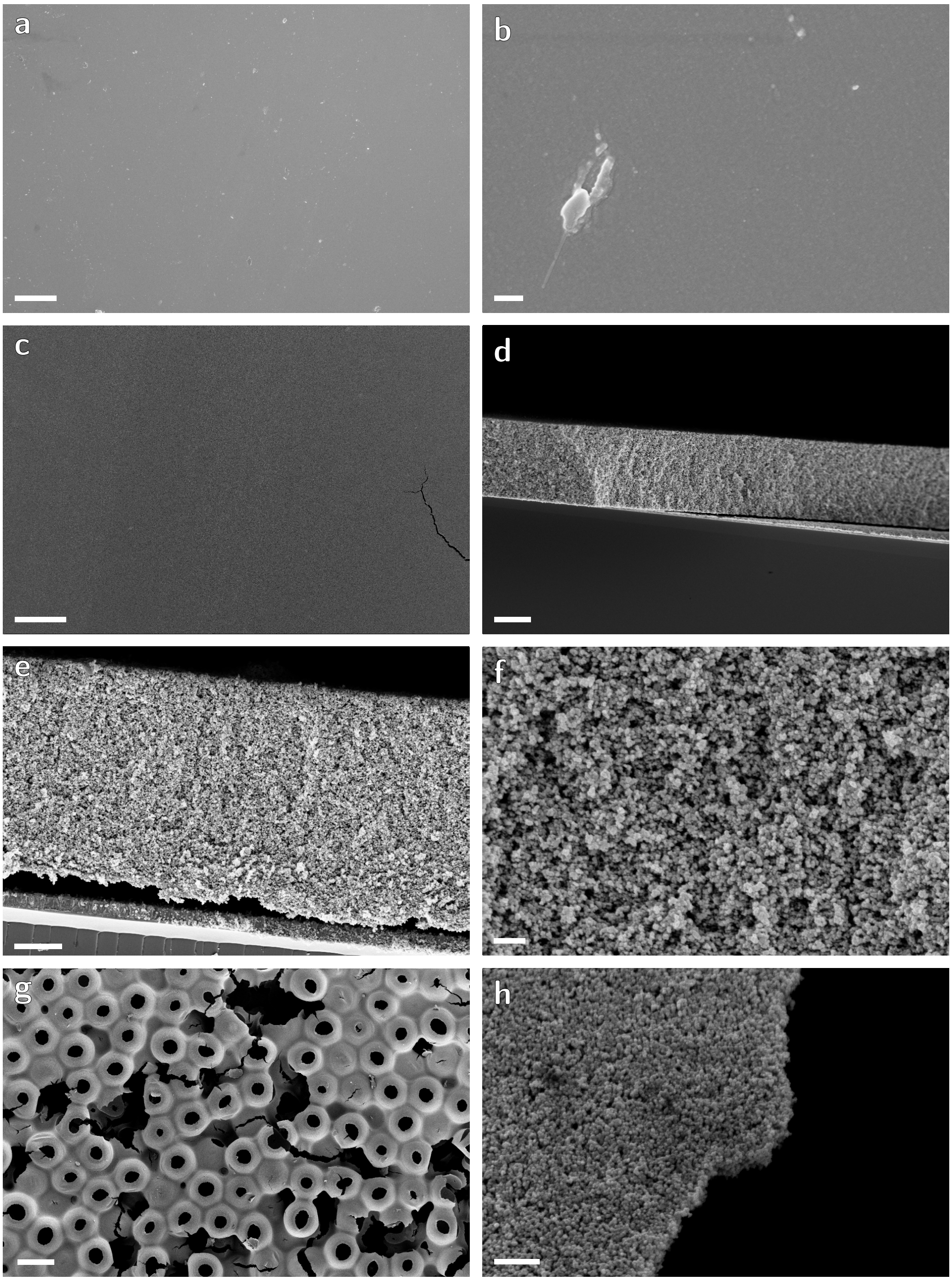

Supplementary Figure 1 | SEM images of the three anode types. (a,b) Commercial non-porous ITO film on PET, with scale bars of 40 μm and 400 nm, respectively. (c) Nanoporous film of sintered ITO nanoparticles, top view, with scale bar of 40 μm. (d,e,f) Cross-section of the nanoporous electrode, scale bars are 4 μm, 2 μm, and 400 nm, respectively; (e) also shows the underlying conducting FTO layer on the glass substrate. (g,h) Top-view of a microporous electrode, with scale bars of 40 μm and 500 nm respectively. The defects in (b) and (c) are non-representative and are shown to visualise the smoothness of the surrounding film.

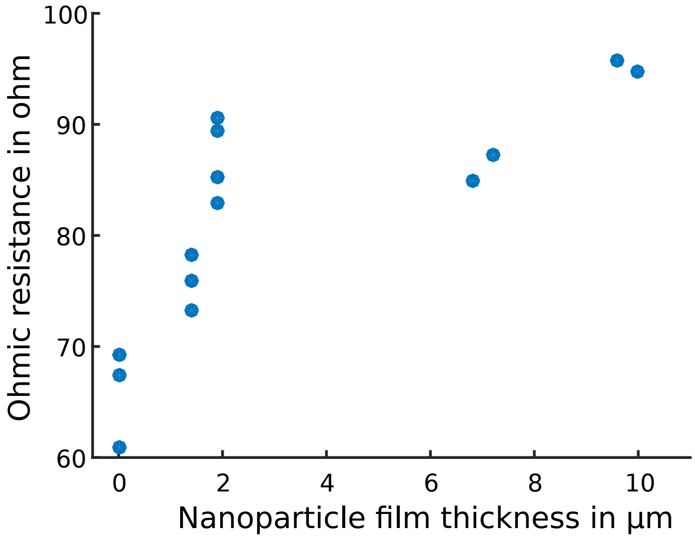

Supplementary Figure 2 | Resistance of different ITO nanoparticle film thicknesses. Data points represent the ohmic resistance (averaged real part of impedance measurement results) of dry nanoporous ITO ‘sandwich’ samples on an ITO glass substrates, for different film thicknesses. Zero thickness refers to a pair of ITO substrates without a sandwiched coating.

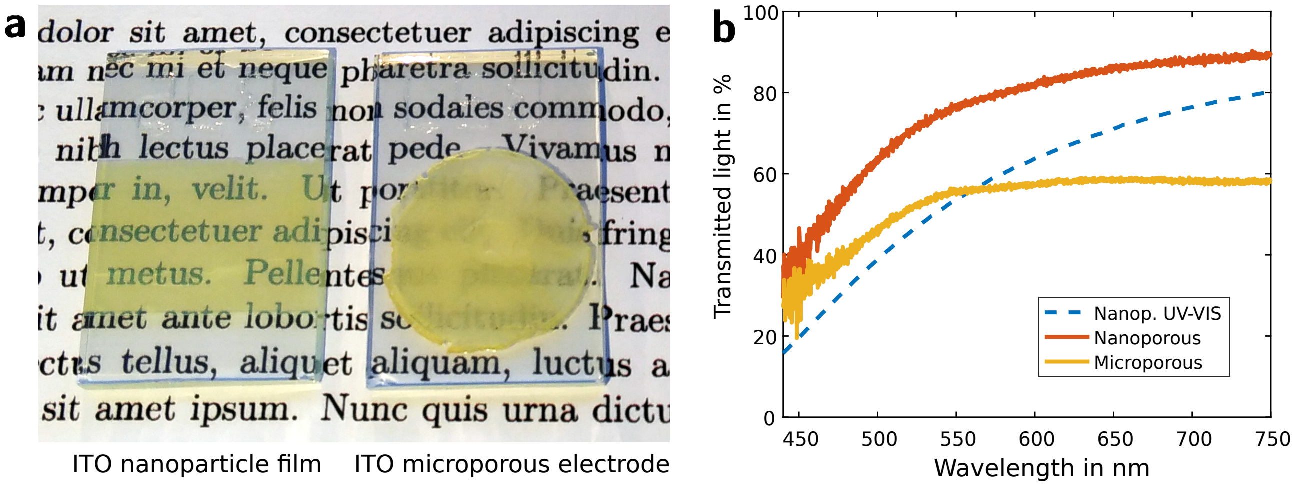

Supplementary Figure 3 | Macroscopic appearance of the translucent electrode material. (a,b) Photographs of nano- and microporous ITO nanoparticle films on FTO-glass substrates, respectively. The microporous structure scatters visibly more light compared to the nanoporous morphology. (b) Transmission spectra of the two electrode types in (a) versus a FTO-glass reference. Integrating sphere measurements record the total transmitted light, while the UV-VIS spectrometer data (dashed line) only captures the light transmitted along the optical axis omitting the scattered transmitted light. The microporous electrode transmits less light because of increased scattering caused by the internal interfaces which leads to increased backscattering.

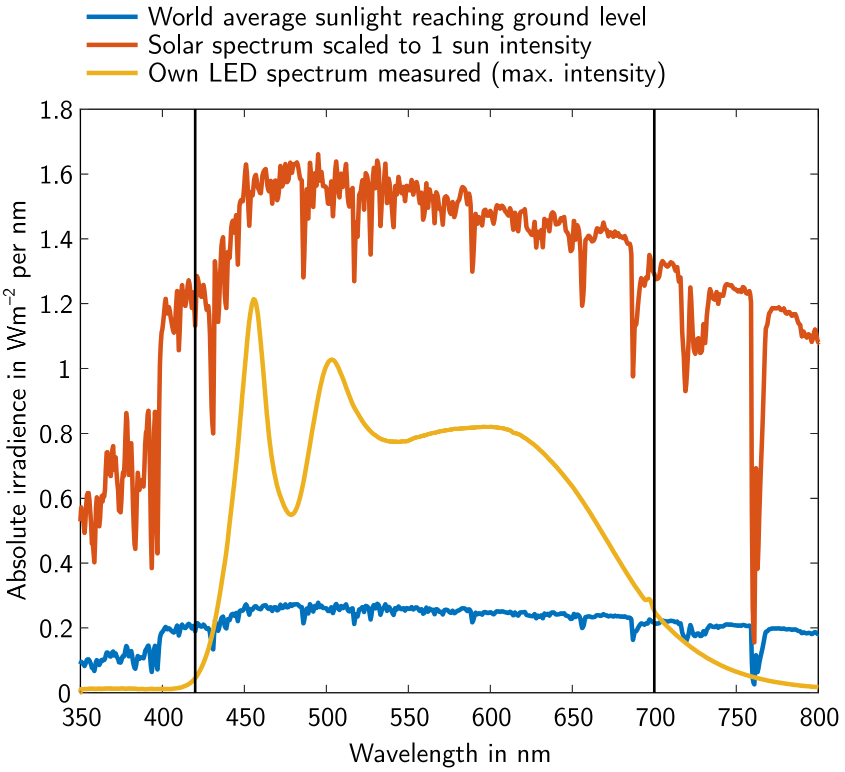

Supplementary Figure 4 | Illuminating light source. Spectrum of custom LED light source (yellow) at maximum intensity compared to the solar spectrum (red) (reference AM 1.5 Spectrum by ASTM, global tilt) at intensities of 1000 W m^{-2} (solar simulator standard) and the world average sun intensity reaching ground level (blue).

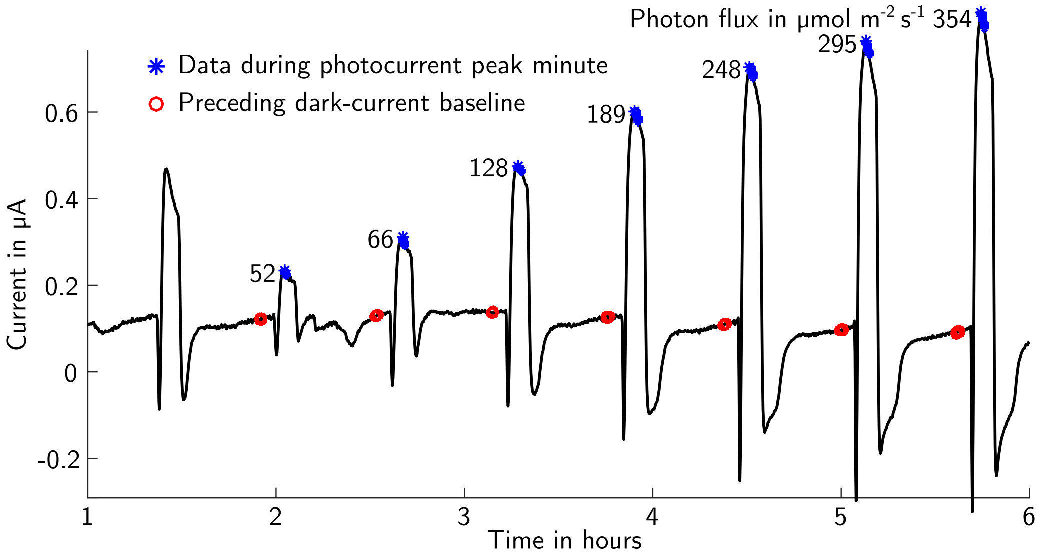

Supplementary Figure 5 | Visual explanation of peak currents. The first seven photocurrent peaks are shown of an illumination sweep with increasing light intensity. The photocurrent peaks are indicated with stars and numbers, along with data points during the peak minute. Averaging peak-minute values provided a reliable value that was less dependent on noise and peak shape. Illumination periods where chosen short enough (5-7 min.) and separated enough (20-30 min.) to let the baseline dark-current recover before the next illumination. To account for baseline fluctuations, the magnitude of each photocurrent peak was measured from the preceding dark-current, here indicated by red circles. The base level was also taken as average of data during one minute. Underlying data is a chronoamperometry measurement fragment of a *Synechocystis* BPV with nanoporous electrode operated at short-circuit.

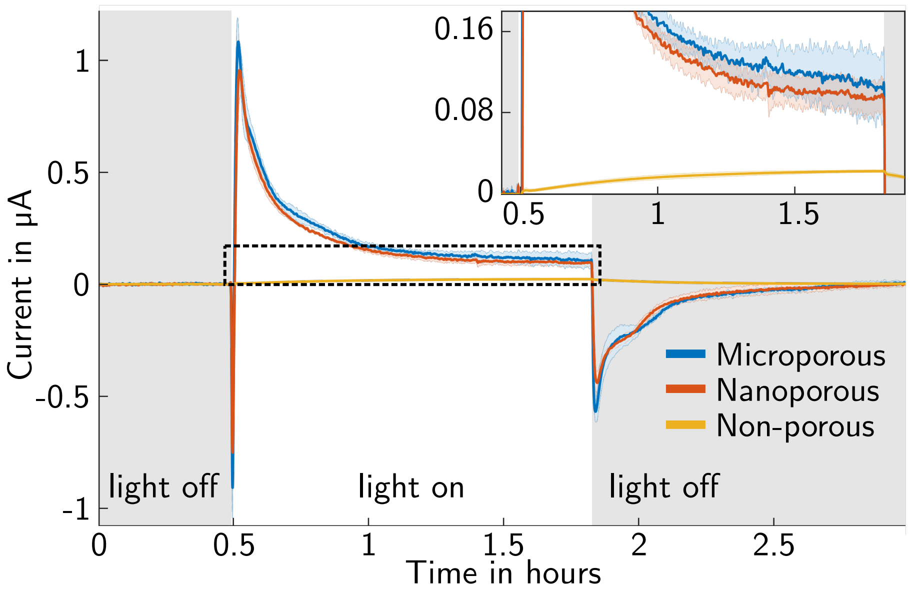

Supplementary Figure 6 | Light response characteristics of the three different *Synechocystis* bio-anode types over time, operated in half-cell mode at 0.2 V vs. an Ag/AgCl reference electrode. The photon flux during illumination was 512 μmol m^{-2} s^{-1}. Averages and standard deviations (shaded) where formed from 3-4 individual devices per anode type.

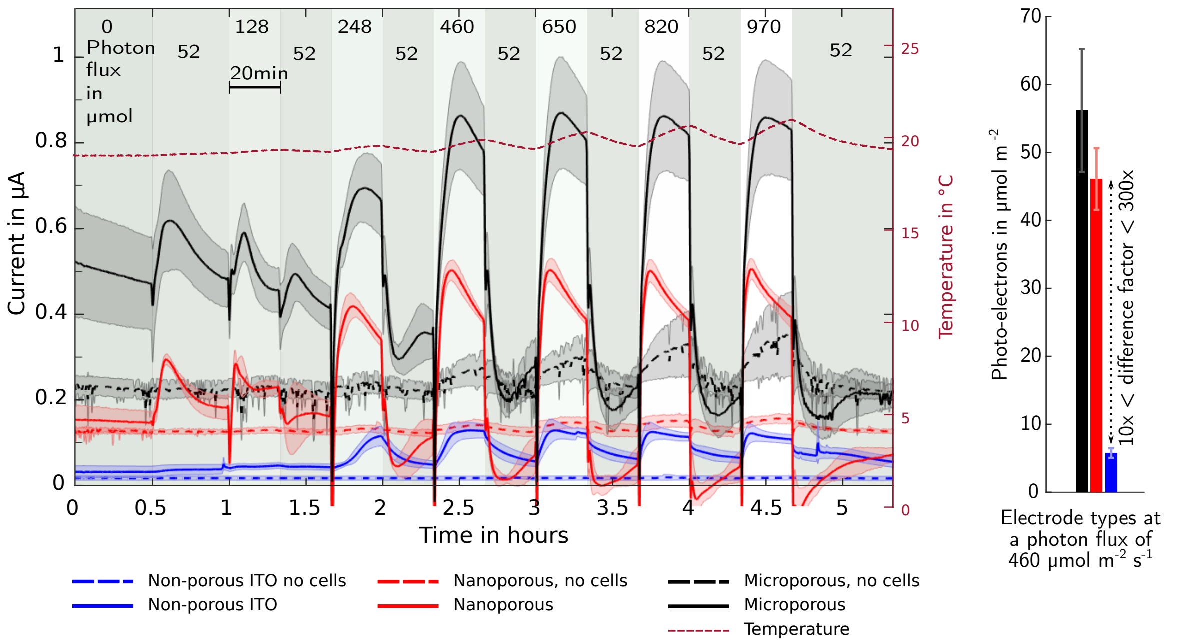

Supplementary Figure 7 | Current generated by electrochemical devices (in half cell mode at 0.2 V vs. Ag/AgCl) with and without biofilms (*Synechocystis*) operated at different light intensities. The shaded regions of the graph represent standard deviations. The electrolyte in the devices was BG11 medium without supplementary phosphate buffer. The temperature curve corresponds to the values measured in the electrolyte close to the anode. In contrast to other measurements presented in this study, the dark-times between illumination times were not chosen to be long enough in this measurement for the dark current level to recover to its pre-illumination base-level.

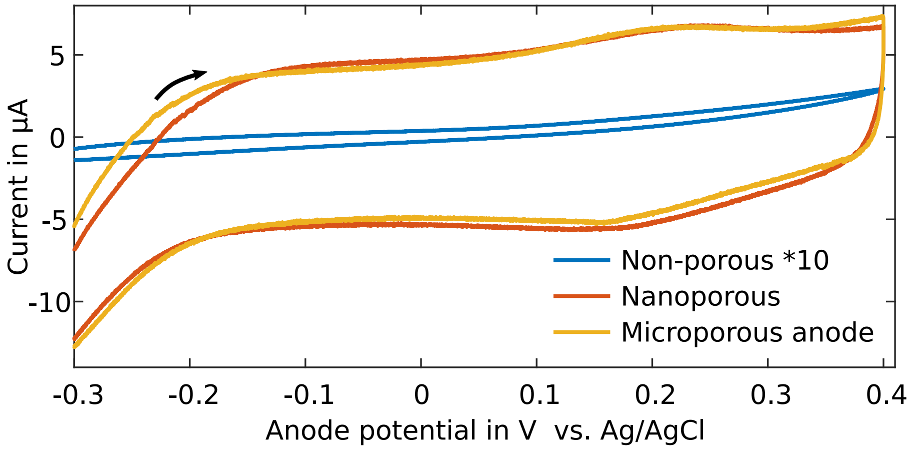

Supplementary Figure 8 | Non-Faradaic charging current on porous electrodes broadens cyclic voltammetry (CV) scans. Example CV data at scan rate of $0.5\,\mathrm{mV\,s^{-1}}$ on *Synechocystis* bioanodes differing in porosity is shown. As indicated by arrows, data from scans performed on porous anodes are broadened in comparison to non-porous electrodes (the later was ten times enlarged for clarity) due to enhanced charging currents. They are also more sensitive to detect electrochemically relevant molecules near the electrode, as described in the results section.

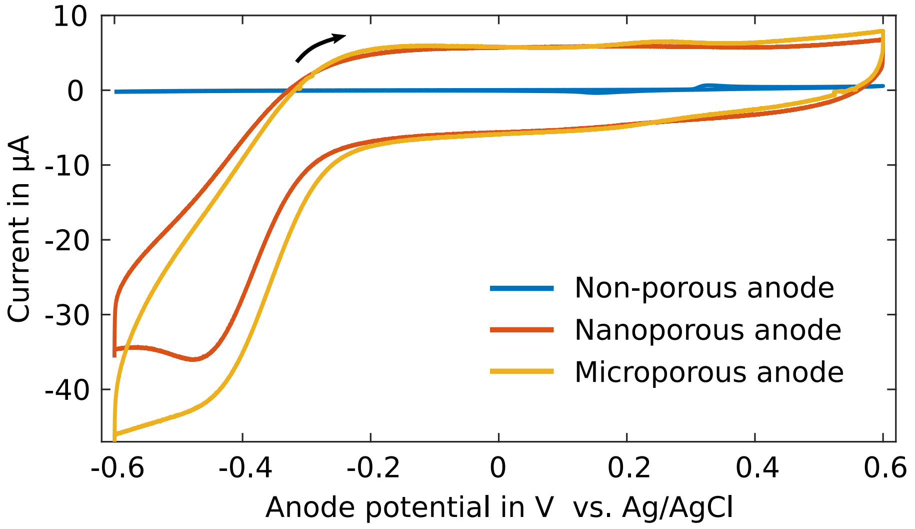

Supplementary Figure 9 | Data demonstrating the extent of surface charging by anode type. Individual cyclic voltammetry scans were taken at a scan rate of $0.5\,mV\,s^{-1}$ with BG11 medium (without microorganisms and light) on anodes differing in porosity. Charging currents were comparable for both porous anode types but much lower for non-porous anodes. These results are expected when taking into account that the microporous anode is made from the same nanoporous material as the nanoporous anode.

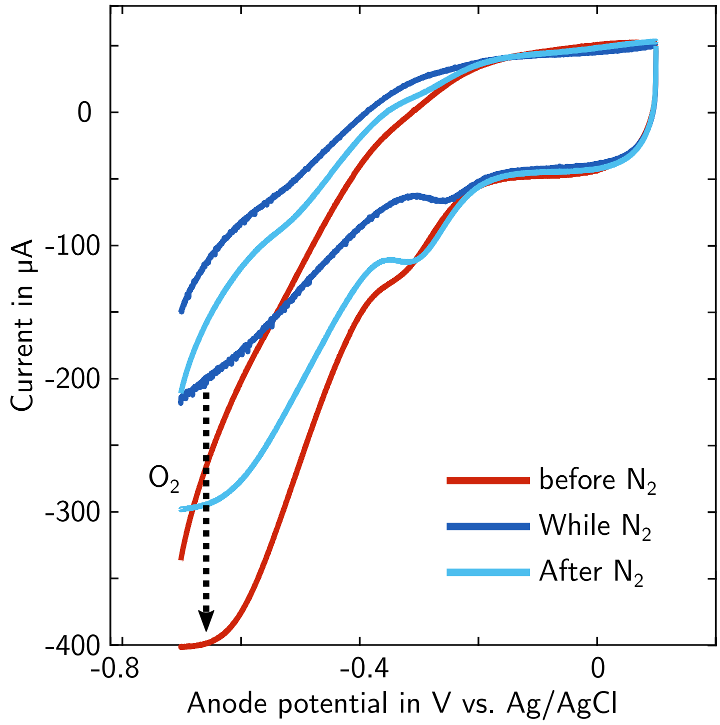

Supplementary Figure 10 | Cyclic voltammetry analysis of a microporous bio-anode with *Synechocystis* cells at a scan rate of 4 mV s^{-1}. Scans from -0.71 to 0.1 V vs. Ag/AgCl at a photon flux density of 295 μmol m^{-2} s^{-1}. The tracks were recorded before, while and after purging the electrolyte with nitrogen gas to remove the oxygen released by the photosynthesising organisms.

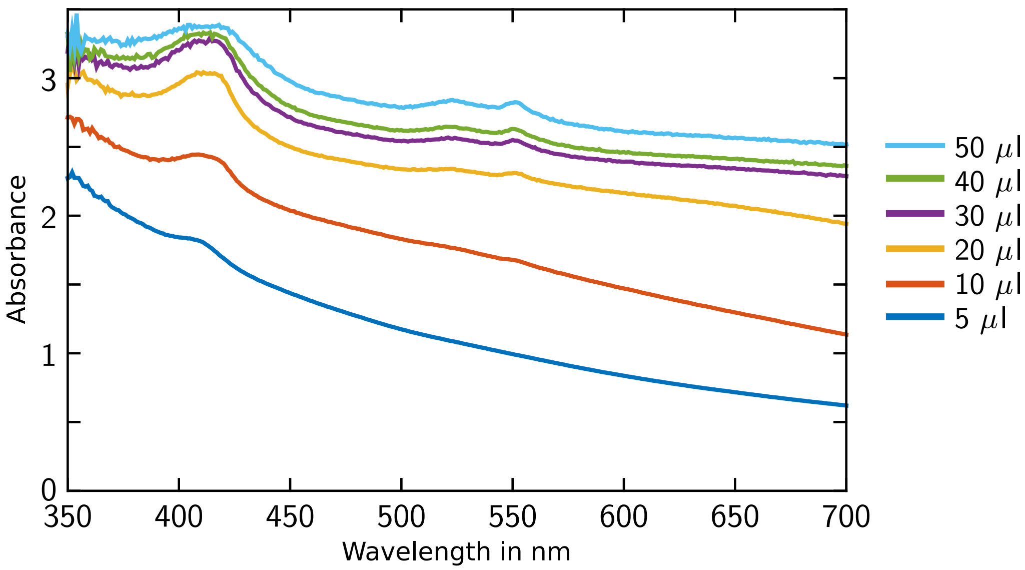

Supplementary Figure 11 | UV-VIS absorption spectra of liquid *Shewanella* cell-culture at different dilution ratios. The amount of concentrated cells between 5 µl and 50 µl, as indicated in legend, was added to 1 ml of LB medium (reference solution) for each measurement. 140 µl of concentrated cell-culture was used per device for electrochemical measurements. This data serves as a reference for the amount of cells added, in absence of an equivalent protocol for the determination of chlorophyll concentration.
